# RNA-Based Sensor Systems for Affordable Diagnostics in the Age of Pandemics

**DOI:** 10.1021/acssynbio.3c00698

**Published:** 2024-04-08

**Authors:** Ilkay Cisil Koksaldi, Dongwon Park, Abdurahman Atilla, Hansol Kang, Jongmin Kim, Urartu Ozgur Safak Seker

**Affiliations:** † UNAM − Institute of Materials Science and Nanotechnology, National Nanotechnology Research Center (UNAM), Bilkent University, Ankara 06800, Turkey; ‡ Department of Life Sciences, Pohang University of Science and Technology, Pohang 37673, South Korea

**Keywords:** RNA sensors, toehold switch, riboregulatory, synthetic biology, cell-free, biosensors

## Abstract

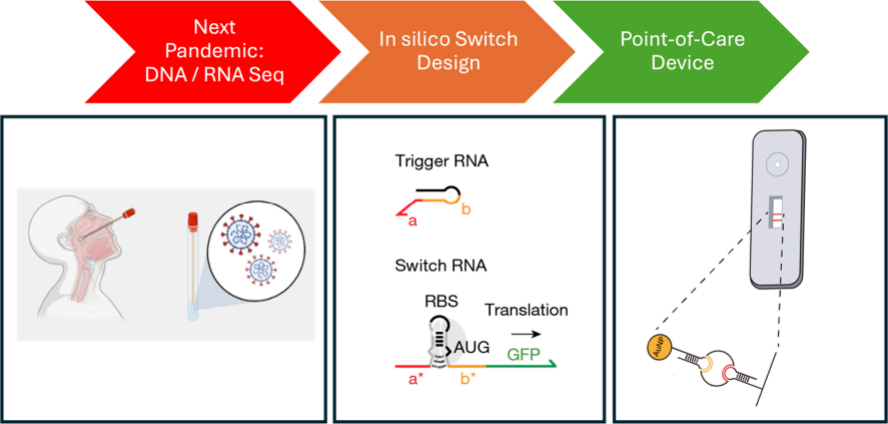

In the era of the COVID-19 pandemic, the significance of point-of-care (POC) diagnostic tools has become increasingly vital, driven by the need for quick and precise virus identification. RNA-based sensors, particularly toehold sensors, have emerged as promising candidates for POC detection systems due to their selectivity and sensitivity. Toehold sensors operate by employing an RNA switch that changes the conformation when it binds to a target RNA molecule, resulting in a detectable signal. This review focuses on the development and deployment of RNA-based sensors for POC viral RNA detection with a particular emphasis on toehold sensors. The benefits and limits of toehold sensors are explored, and obstacles and future directions for improving their performance within POC detection systems are presented. The use of RNA-based sensors as a technology for rapid and sensitive detection of viral RNA holds great potential for effectively managing (dealing/coping) with present and future pandemics in resource-constrained settings.

## Introduction

Over the past 50 years, there have been several outbreaks of infectious diseases such as Ebola, Zika, SARS, MERS, and, most recently, COVID-19. These outbreaks have caused significant losses and have put a burden on healthcare and economic systems, highlighting the urgent need for efficient, accurate, affordable, and easy-to-use technologies for on-site pathogen detection. To address this need, innovative molecular biology methodologies, such as qRT-PCR, lateral flow immunoassays, enzyme-linked immunosorbent assays (ELISA), DNA microarrays, fluorescent in situ hybridization (FISH), and RNA-based sensors, have been developed. Among these, the toehold switch-based system stands out as a promising technique for its capacity to detect and respond to target RNA sequences selectively.

To address the need for point-of-care (POC) devices with simple field operation capacity and no requirement for intensive equipment or qualified personnel, innovative approaches have been developed. One significant example of such approaches is the toehold-based POC diagnosis systems.^1,2^ The toehold switch sensors adapted to paper-based cell-free platforms simplify distribution and utilization in low-resource settings while being inexpensive per test, rapid and highly sensitive, and specific.^3,4^ In light of recent remarkable developments in the field, we summarize the operation and design principles of riboregulators, followed by the most recent applications and current challenges of the system.

## Advantages of RNA as a Building Block of a Synthetic Circuit

RNA molecules play essential roles in cellular networks such as templates for translation, regulators to control gene expression levels, and sensors to perceive external signals.^1,5,6^ Due to these characteristics and the ability of RNA molecules to provide regulation at the transcription or translational level, RNA has been widely used as biological circuit elements in synthetic biology applications.^7^ Advancements in RNA synthetic biology led to the development of RNA circuits to perform unnatural functions such as logic operations, sensors to detect small molecules, and cancer cell identifiers.^7−9^

## General Descriptions of a Riboregulator

Riboregulators are endogenous RNA molecules that modulate gene expression at the post-transcriptional level by responding to environmental cues or other RNA molecules.^10−17^ Riboregulators can act as sensors, regulators, or effectors of gene expression by altering the accessibility, stability, or activity of target RNAs.^18−20^ Riboregulators can operate through various mechanisms, such as base pairing, folding, cleavage, or modification of target RNAs.^21−23^ Furthermore, they can regulate gene expression either negatively or positively depending on the context and design of the system. Riboregulators play important roles in various cellular processes such as development, stress response, and pathogenesis.

Riboswitches that are naturally present in cells have evolved to perform specific functions. Though, synthetic biology has enabled the programmable design of riboswitches that act as activators or repressors in response to desired RNA sequences. Similar to endogenous riboswitches, toehold-based switch sensor systems are synthetic RNA devices that regulate gene expression at the translational level in response to specific RNA triggers. These systems consist of a toehold domain that can hybridize with a complementary trigger RNA sequence and a stem-loop structure that sequesters the ribosome binding site of a reporter gene. In the absence of the trigger RNA, the toehold switch is in an OFF state and prevents translation of the reporter gene. When the trigger RNA is present, it binds to the toehold domain and disrupts the stem-loop structure, exposing the ribosome binding site and allowing the translation of the reporter gene. This mechanism enables selective and programmable detection and response to target RNA sequences in various biological contexts. Moreover, toehold-based switch sensor systems can implement multiple logic gates, enhancing their functional diversity.

## Riboregulators and Toehold Switches

### General Description of a Synthetic Circuit and Limitation of Conventional Genetic Devices: Advantages of RNA-Based Circuits

RNA-based genetic circuits have emerged as a promising tool in synthetic biology due to their unique advantages over protein-based circuits. One of the key benefits of RNA-based circuits is their ability to be designed de novo, meaning that they can be created from scratch using computer-aided design software. This approach allows for greater flexibility in circuit design and reduces the time and cost associated with traditional protein engineering methods. RNA-based circuits are encoded with a lower cellular load compared to protein-based circuits, as RNA molecules are simpler in structure and easier to synthesize. Another advantage is that RNA circuit components can be colocalized within the cell, which allows for self-assembly into functional complexes, unlike proteins which often require specific protein–protein interactions. Also, synthetic RNAs are less likely to interfere with the normal functioning of the host cell, unlike proteins.

RNA-based circuits have several examples, including the incoherent feedforward loop (IFFL) circuit that allows for precise temporal control of gene expression,^24^ the exclusive OR (XOR) circuit that can function as a biological switch by controlling the expression of two genes in a mutually exclusive manner,^25^ and the cell-free coherent feedforward loop (CFFL) circuit, which can operate outside of living cells and have potential applications in biotechnology and medical diagnostics due to its potential to filter out noise in gene expression via reducing leakage and increasing the fold-change of the output in synthetic genetic circuits.^26^ Overall, RNA-based genetic circuits have numerous advantages over protein-based circuits, including better design flexibility, reduced cellular load, and the ability for self-assembly, making them a promising tool for synthetic biology research and applications.

**Figure 1:**
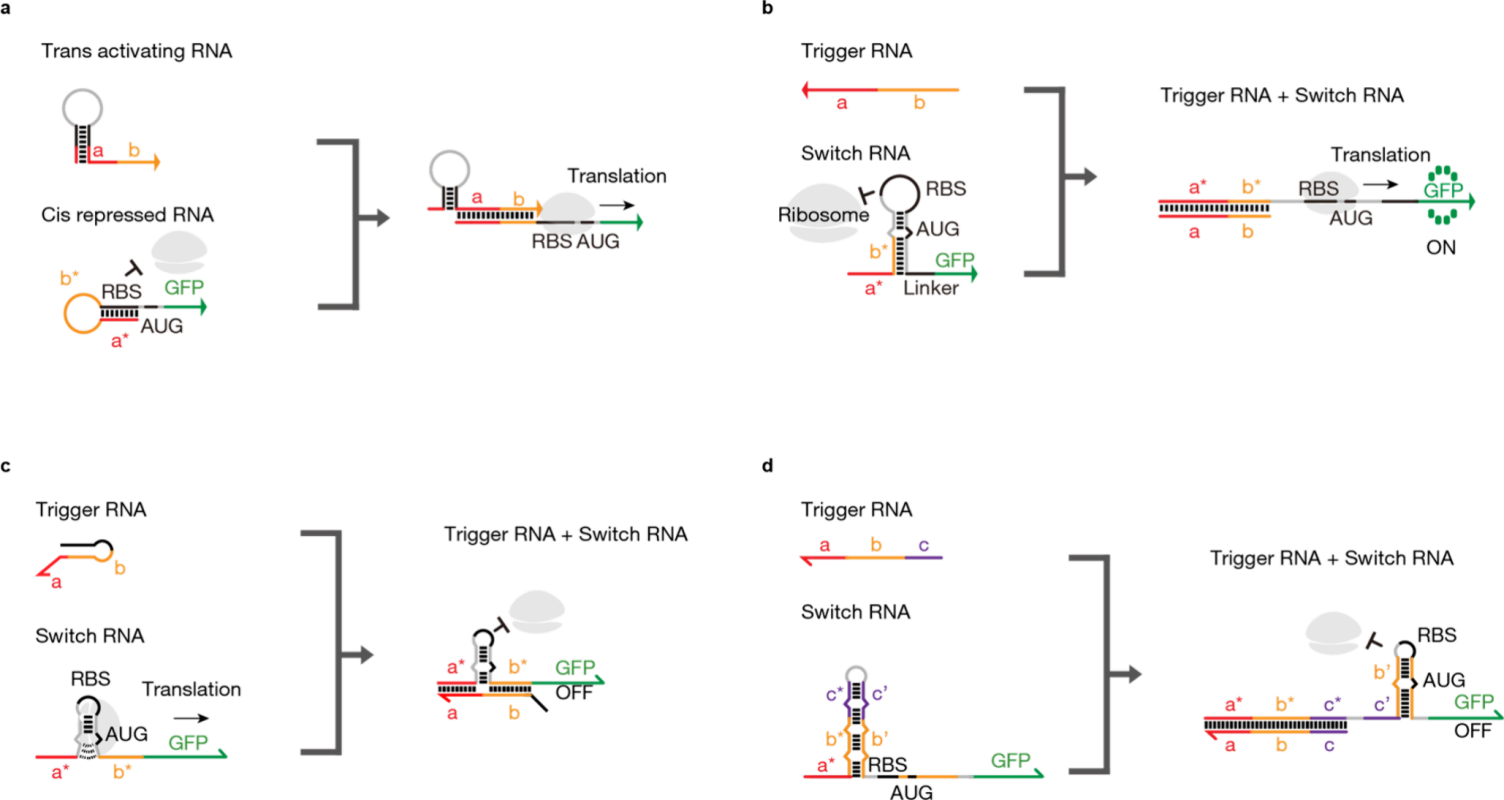
Mechanisms of the Action of Various Riboregulators. (a) Conventional riboregulator. (b) Toehold switch. Figure adapted from ref (30). Copyright 2014 Cell Press. (c) 3WJ repressor. Figure adapted from ref (36). Copyright 2019 Nature Publishing Group. (d) Toehold repressor. Figure adapted from ref (36). Copyright 2019 Nature Publishing Group.

Different types of riboregulators and their distinct mechanisms of action to regulate gene expression at the post-transcriptional level are illustrated in Figure 1. The first example shown in Figure 1a depicts the conventional riboregulators, which operate by binding to a small molecule, like a metabolite, and then either promoting or inhibiting downstream gene translation. The toehold switch shown in Figure 1b functions by binding a small RNA molecule that is complementary to its own sequence and activates gene translation. The 3WJ repressor forms a hairpin-like structure to halt the downstream gene translation, as shown in Figure 1c. The toehold repressor is similar to the toehold switch; however, it acts as a repressor upon binding to its complementary small RNA molecule and sequesters the mRNA, as shown in Figure 1d. Their mechanisms of action can be finely tuned to achieve precise control over the gene expression. These different types of riboregulators offer a range of design options for synthetic biology applications and beyond.

Riboswitches are natural RNA sensors that can control transcription and translation of mRNA due to conformational changes upon ligand binding in the same RNA that encodes the gene. Several natural riboswitches that detect various small molecules to regulate numerous numbers of genes have been identified both in prokaryotic and eukaryotic organisms.^1,27,28^ Toehold switches, on the other hand, are de-novo-designed riboregulators that control gene expression via base pairing with target RNA sequences.^29,30^ These RNA-based sensory and regulatory systems have gained scientific interest due to their capacity to be engineered as sensors for various applications, both in vivo and in vitro. Combined with cell-free protein synthesis systems, engineered RNA switches demonstrated the effectual dynamic range and orthogonality as genetic circuit elements.^25,26^ Even though several outstanding riboswitch and toehold-based sensors have been developed with low crosstalk, high efficiency, and low background activity; design and validation are still challenging due to variability in function.^9,27^

Synthetic biology has several challenges in the design of orthogonal, easily manipulated, and analyzed genetic circuit elements for various operations. Since the design of the first genetic circuits, scientists were looking for strategies to enhance the dynamic range of response elements to a target, as well as their orthogonality and metabolic load caused by these designs.^31^ Early genetic circuits were designed with inspiration from natural genetic control mechanisms of protein–DNA interactions. Regardless of the function, control of metabolic production was highly susceptible to interruptions by complex cellular machinery.^32^ To obtain sophisticated sensing machines and complex genetic designs, scientists needed to build advanced circuit elements that could overcome these problems. De-novo-designed riboregulators using Watson–Crick base pairing rules and thermodynamic modeling have been engineered for the purpose of allowing RNA-based genetic elements to acquire more stable and less complex designs.^33^ As a general principle, de-novo-designed riboregulators are composed of a control element harboring the genetic control system of a target gene and an inducer or target element for the activation or deactivation of the production system.^34,35^ By controlling the production and existence of either of these elements, scientists have successfully developed biological sensors that can achieve very high dynamic ranges for desired elements.

The first generation of de-novo-designed riboregulators started by repurposing natural systems that are used by organisms for metabolic control. Later, in the pioneering study of Alexander Green, loop-linear interactions of designed cis-acting (caRNA) and trans-repressing (trRNA) riboregulator RNA pieces were built as an advanced version of riboswitches that can regulate transcriptional output.^30^ In this type of design, the ribosome binding site (RBS) of a promoter-like domain is insulated by pairing sequence constraints and a loop sequence to disable RBS without the existence of a trigger. When taRNA is present, this insulation loop structure is linearized and the RBS is revealed for the expression of the downstream gene. Due to insufficient dynamic range and stability problems of the initial design, the second generation of riboregulators is engineered. In this design, later named as toehold switch, RBS is placed in the loop region, and the triggering RNA is designed for opening only the toehold-like section of the switch element.^36^ With independence from the RBS sequence in the hairpin structure, any RNA sequence could be used to design riboregulators. Moreover, high stability and high dynamic range systems were obtained for various applications. Besides, by using a three-section design for toehold switches, “trigger-off” systems are also designed for the deactivation of riboregulators in the presence of a trigger sequence. As the last type of de-novo-designed riboregulators, 3-way-junction (3WJ) is designed to obtain a more stable structure and a high ratio of successful designs that can achieve higher fold changes.^36^ In this strategy, the triggering sequence also has a loop structure that ensures stability, and by blocking the concealing of switch RNA, it achieves strong repression that is very useful for designing complex circuits.

## Challenges in Building Riboregulators (Leakage, Crosstalk, Laborious, etc.)

Riboregulators have significant potential for use in synthetic biology and biotechnology, but their design and implementation are not without challenges. One of the main challenges is leakage; in this case, the toehold system is unintentionally active or repressed in the absence of the trigger sequence. Leakage can reduce the dynamic range and sensitivity of the riboregulator system and can lead to metabolic burden and toxicity to the host cell.

Another challenge is posed by the sequence constraints; since the self-assembly of components requires base pairing, the process is sequence-dependent resulting in additional residues on the output protein’s N-terminus in the prospect of interfering with its function. Besides this intrinsic, potentially adverse but avertible, feature of the system, there are several cruxes to be considered while constructing de-novo-designed riboregulators. The switch component of the de-novo-designed riboregulators should be stable enough to protect the hairpin loop structure on its own to prevent leakage and background. Still, the energy values should be selected so that it is more favorable to create the trigger-switch complex via base-pairing by opening up the hairpin loop structure in the presence of a trigger. To avoid premature transcription termination of the downstream gene, the switch sequence should not contain intragenic stop codons. Furthermore, the trigger sequence should be selected to be unique to its cognate switch to prevent off-targets. Previous experiments have shown that in silico base-pairing interaction prediction can help filter out candidates with off-target interactions and to prevent crosstalk among multiple de-novo-designed riboregulators.

The other type of challenge is the RNA stability and sample processing. Ribonucleases have the capacity to degrade RNA, which can reduce its stability and usefulness. The functionality of RNA-based devices and treatments may be impacted by this, which can happen in both cellular and noncellular settings. The molecular structure of RNA molecules must be preserved for them to function. However, the stability of RNA structures can be impacted by environmental factors including pH and temperature.^105^ To ensure stability and consistency in the reaction, it may be considered to implement the production of a portable device, as reported in.^117^

The design and optimization of riboregulators require extensive experimental testing and fine-tuning of various parameters such as sequence, length, structure, and location of the riboregulator elements. Moreover, the higher the number of interacting RNA components, the more laborious the challenge of self-assembly and interaction prediction becomes. For instance, as of today, the most complex de-novo-designed RNA circuitry has twelve input units placed in a single layer to perform complex logic computation; though a decrease in signal level and leakage in the false state has been reported.^37^ Compression of de-novo-designed riboregulator elements in a single transcription layer results in minimized delay and an increase in signal propagation while a reduction in protein load and genetic footprint since the process only involves post-translational regulation of gene expression without any transcript or protein intermediates.^38^ These challenges limit the scalability and robustness of riboregulator systems, necessitating the development of novel strategies to overcome them.

## Modeling

RNA structure is a key determinant of its diverse biological functions.^39−42^ Therefore, creating RNA molecules with specific structures and functions is of great value to research, where computational methods for modeling RNA secondary structures come in handy. Thus far, this approach has been utilized for synthetic biology and biotechnology applications to design RNA-based devices, including riboregulators,^3,4,43−45^ aptamers,^46−50^ and CRISPR guides.^51−56^ Current advanced approaches for in-silico RNA design rely on free energy minimization algorithms, which estimate the thermodynamic stability of different structures.^57−59^ To enhance the prediction accuracy, some methods also incorporate experimental mapping data or structural conservation information from a set of homologous sequences.^60−62^ Several prominent software tools have been developed for in-silico RNA design based on these approaches, including but not limited to NUPACK, RNAstructure, ViennaRNA, UNAfold, and the deep-learning framework. These tools offer a variety of features, such as analysis, prediction, comparison, and synthesis of nucleic acid structures and interactions, each with its own advantages and drawbacks.

**Figure 2:**
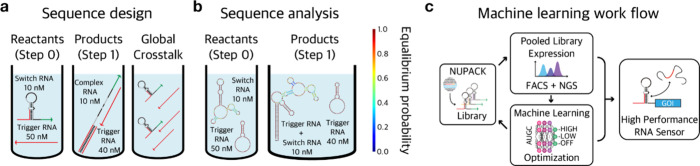
Schematic diagram for RNA molecule modeling and analysis using NUPACK. (a) Schematic diagram for riboswitch sequence design regarding cognate interactions and global crosstalk. Figure adapted from ref (119). Copyright 2019 The American Chemical Society. (b) Schematic diagram for riboswitch sequence analysis regarding reactants and products. Figure adapted from ref (119). Copyright 2019 The American Chemical Society. (c) The workflow when NUPACK was used with machine learning technique.

NUPACK software provides algorithms to perform the complex design of RNA structures, RNA-RNA interactions, multitube design,^63^ and test tube design,^64^ which is available as a Web site interface and Python module.^57^ RNAstructure software offers RNA secondary structure prediction, analysis, and RNA sequences that fold into a predefined structure with a graphical user interface with a platform-independent compilation.^65^ The ViennaRNA Package is a C code library and a Web site interface to predict RNA secondary structure via energy minimization and to design RNA with a predefined structure.^58^ UNAfold software is a command-line-oriented tool to predict nucleic acid folding and hybridization and to perform inverse and constrained folding.^59^ The deep-learning framework has been utilized to optimize toehold sequences with NuSpeak and STORM pipelines, where the latter conserves partial trigger sequence, and the former allows complete redesign.^66^ Deep neural networks have been employed to predict the function and design toehold switches targeting human transcription factors and several viral genomes.^67^ An example workflow for toehold design utilizing NUPACK and a machine learning approach can be seen in Figure 2.

The design and prediction of the behavior of engineered RNA switches are challenging due to poorly understood design rules. In this prospect, computer-based RNA structure prediction software have been applied to accelerate the massive identification of RNA switches with diverse functions and applications.^30,36^ For instance, toehold switches adopted NUPACK for massive in silico design and validation of switches (Figure 2a). Their pairwise interactions were simulated to evaluate cross-talk interactions, which were impossible without structure prediction software (Figure 2b). This approach was applied for the detection of pathogenic bacteria and viral RNA such as Zika, Ebola, HIV, and SARS-CoV-2.^3,4,45,68,69^ Still, the validation and screening of RNA switches were time-consuming, limiting their quick application for pandemic virus diagnostics.

To enhance the efficiency of RNA switch design, machine-learning approaches have been integrated with computer-aided RNA structure prediction and design (Figure 2c). For example, machine learning algorithms such as random forest have been used for RNA target selection algorithms, which enabled the design of RNA switches with improved sensitivity and specificity for single-stranded target RNAs.^66,67,70^ Furthermore, machine learning algorithms also enabled the design of improved analyte sensors which are better at sensitivity or specificity.^71^

Nowadays, multiple software such as NUPACK,^57^ RNAstructure,^72^ RNAfold,^58^ or Kinfold^73^ to predict and score RNA structures have been used as input to deep learning models.^66,67,74^ These approaches are highly effective in automating RNA switch design and characterizing crucial factors of RNA switch efficiency. The resulting method achieved large, statistically significant improvement in predicting noncanonical and non-nested base pairs, which are planar hydrogen bonded pairs of nucleobases that differ from the standard Watson–Crick base pairs.^75,76^

In addition to predicting RNA structures based on the thermodynamic approach, it is becoming more important to count on kinetic effects (reaction velocity) for precise predictions of RNA interactions. This was experimentally demonstrated in the case of predicting TMSD (toehold-mediated strand displacement) reactions through thermodynamic software without a kinetic trap.^113^ Several groups have reported RNA interaction analysis based on current principles of kinetics and simulation tools. In 2014, the Louis group introduced the coarse-grained RNA model, oxRNA, at the nucleotide level and applied it to a kinetic model for RNA TMSD in 2015.^114^ These early RNA TMSD kinetic simulations have evolved with a study predicting the performance of a TMSD RNA sensor through simulation.^115^ However, it should be noted that there are regrettable absences of several experimental evaluations in the kinetic simulation of RNA interactions. In 2021, the Keyser group experimentally demonstrated kinetic changes in the kinetics of DNA TMSD reactions in a metal–organic cage (Fe^II^_4_L_4_) environment.^122^ This suggests that the kinetics of RNA TMSD reactions can also vary depending on the surrounding environment. Additionally, a study by the Simmel group reported in JACS in 2022 conducted kinetic simulations and experiments on the reactivity of DNA TMSD in a random sequence DNA pool, emphasizing once again the impact of the surrounding environment on the reaction.^118^ RNA’s wobble base pairing, non-Watson–Crick base pairing, and tertiary structure interactions provide somewhat restricted approaches to kinetic models. Further advances in understanding RNA kinetics may enable predictions of the thermodynamic and kinetic performance of TMSD RNA sensor in various environments.^116^

## Application

**Figure 3:**
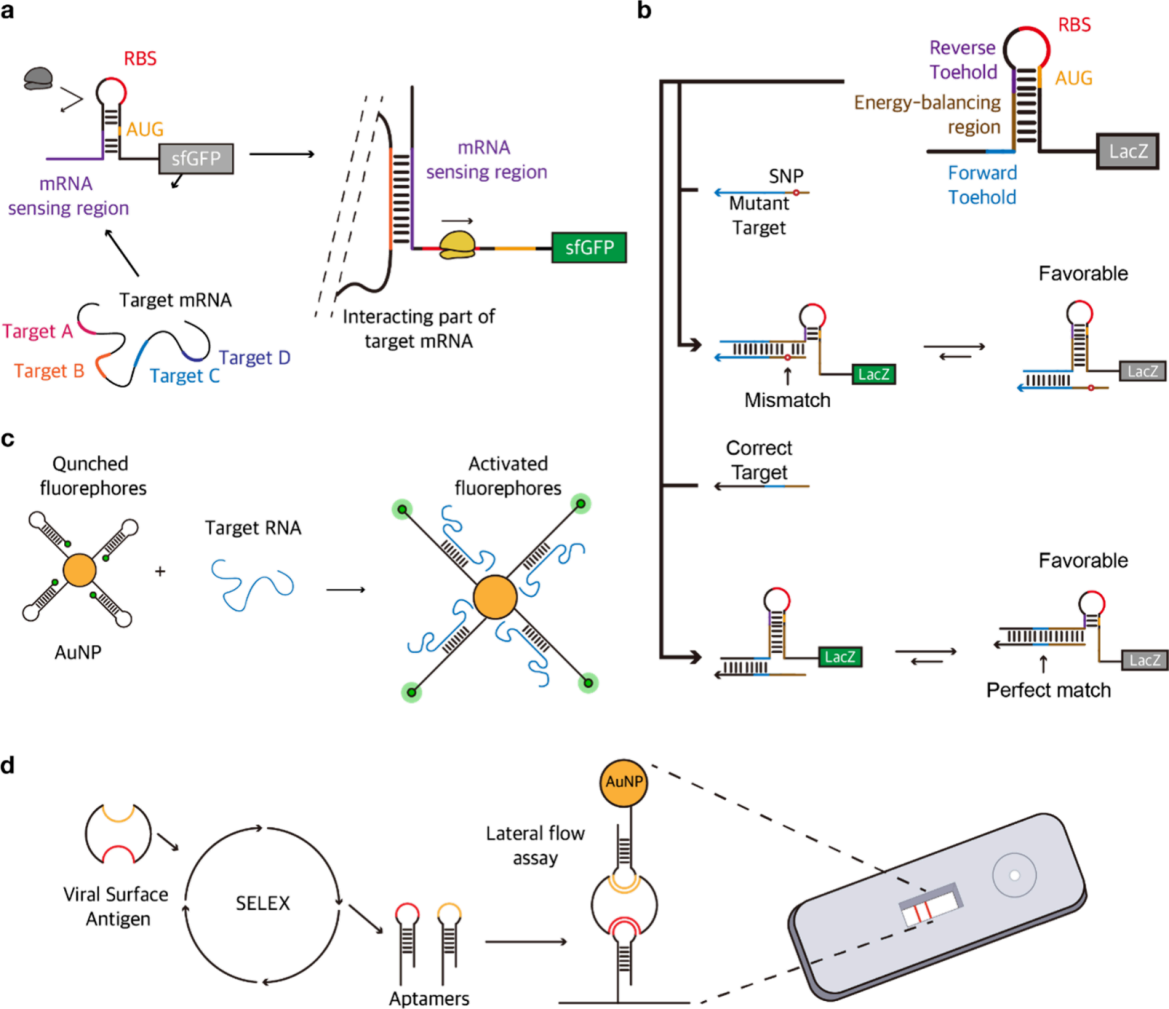
(a) A schematic of toehold switch-based mRNA sensing diagnostics. (b) Working mechanism of toehold switch-based SNP detector. Figure adapted from ref (81). Copyright 2020 Cell Press. (c) A molecular beacon-based viral RNA or miRNA sensing platform. Figure adapted from ref (120). Copyright 2012 The Royal Society of Chemistry. (d) SELEX-based viral antigen-sensing aptamer engineering workflow.

De-novo-designed riboregulators, specifically toehold switches, have demonstrated potential in diverse applications: gene expression regulation, endogenous RNA sensing, detection of biomarkers and pathogens, bioproduction optimization, guided stem cell differentiation, distinguishing cell types and states, and development of gene therapy. Although there are sequence restrictions in the toehold design that have yet to be advanced, these studies highlight the significance of in silico design tools for optimization steps (Figure 3).

Toehold switches can regulate the expression of genes of interest by responding to endogenous or exogenous RNA signals. This can enable the study of gene function, regulation, and interaction in different biological systems. For instance, toehold switches have been utilized to control endogenous gene expression in bacteria,^30^ yeast,^77^ and mammalian cells.^78^ Furthermore, independent regulation of twelve genes’ expression or endogenous RNA sensing have been established in bacteria by Green et al.^37^

By sensing the levels of RNA transcripts or molecules involved in the biosynthetic pathways, we can modulate the biosynthesis of metabolites or proteins. This approach can enable the optimization of bioproduction or the engineering of novel biosynthetic functions. toehold switches have been used to regulate the production of 3-hydroxypropionic acid, violacein, and lycopene in bacteria.^79^

For various biotechnological applications in eukaryotic cells, the ability to control gene expression is crucial. However, low-fold changes in gene expression and the size of trigger RNAs are current limitations. Zhao et al. introduced a modular eukaryotic riboregulator system, eToeholds, to control endogenous or exogenous RNA translation. This approach enabled discrimination between different cell types or states among different viral infection statuses by sensing the expression of specific genes or factors characteristic of a certain cell identity or condition and activating the expression of genes that mark or modulate that cell type or state.^77^ This approach can enable the identification or manipulation of target cells for therapeutic or research purposes. Toehold switches can bind to specific biomarkers, such as metabolites or RNA molecules associated with certain diseases or conditions, and trigger protein production that can serve as a diagnostic marker. To date, toehold switches have been used to detect infectious pathogens, such as bacteria or viruses. Takahashi et al. have developed a highly specific tool to analyze biomarkers of ten different species in gut microbiome with the capability of mRNA quantification comparable to that of quantitative polymerase chain reaction (qPCR) in addition to the diagnosis of *Clostridium difficile* infection.^80^ Several groups around the world developed toehold-based sensors for virus detection, including Zika,^3,69^ Ebola,^4^ SARS-CoV-2,^45^ HIV^68^ viruses.

During the Zika outbreak, Pardee et al. integrated toehold sensors with a cell-free and paper-based system for low-cost and portable detection of the target virus.^3^ The implementation proved that de-novo-designed riboregulators can be used as POC diagnostic devices. Even though the diagnostic platform applies to clinical samples, the dilution requirement leads to lower sensitivity, which can be compensated for by extending the nucleic acid amplification duration. With this approach, a specific and sensitive platform for nucleic acid detection has been achieved, which can be used in remote areas to mitigate the risk of infection without the need for specialized personnel or equipment, unlike standard testing procedures such as qRT-PCR or ELISA. Furthermore, Hong et al. have engineered toeholds that can sense single-nucleotide polymorphisms (SNPs), namely SNIPR.^81^ With single-nucleotide resolution, mutations or virus strains can be detected with ease without the need for a sequencing step.

Also, for the clinical validation, Saxena et al. used RNA samples from COVID-19 patients to utilize the electrochemical sensor and the lateral flow dip strips. The findings demonstrated that although the multigene approach enabled positive detection utilizing more target areas, the lateral flow dip strips produced false-negative results for two samples. The designed assays, such as the electrochemical sensor for N-gene and lateral flow device (LFD)-based RT-LAMP test, showed agreement with RT-PCR findings, suggesting their potential for a precise and sensitive diagnosis of COVID-19, particularly in the setting of developing variants with numerous mutations.^100−102^

Once the application is looked at, FDA-approved RNA sensors are shown. Luke et al. have investigated nitazoxanide (NTZ) as a potential oral therapy for Ebola virus (EBOV) infection. One of the RNA sensors employed in this work is the RIG-I-like receptor (RLR) pathway. According to the research, NTZ treatment increases RLR activation in response to the cytoplasmic dsRNA stimulation, which causes an increase in interferon activity and initiates the antiviral phosphatase reaction.^103^ However, the development of FDA-approved RNA sensors with CRISPR is ongoing. Liu et al. have investigated the utility of CRISPR/Cas systems as biosensors for nucleic acid detection. Additionally, the study investigates the possibility of repurposing CRISPR/Cas systems from genome-engineering applications to create practical and efficient instruments for nucleic acid detection, with a focus on their application in point-of-care testing (POCT) devices.^104^ Currently, the only FDA-approved nucleic acid sensors based on synthetic biology for COVID-19 are SHERLOCK and DETECTOR (Revoked), both of which have Emergency Use Authorization (EUA). The existence of synthetic biology diagnostic technologies is limited to sporadic reports, raising concerns about consistency and reproducibility. The two technologies granted EUA primarily involve target nucleic acid amplification through Recombinase Polymerase Amplification (RPA) and cleavage of reporter probe DNA (quencher and fluorophore) using Cas12 or Cas13. Both RPA and probe DNA have been commercialized, ensuring consistency and reproducibility through prolonged use. Moreover, during the commercialization process, these products have integrated lateral flow assay-based reporting methods, making them akin to rapid antigen tests in terms of rapidity. When benchmarking, the toehold switch approach of CFPE should ensure consistency and reproducibility through various tests, and applying lateral flow assay, instead of the conventional colorimetric method using LacZ, would reduce heterogeneity, aligning it with established rapid antigen testing methods

The toehold design is sequence-dependent, though the detection platform is not; although, for an unknown sample, sequencing is required. Wang et al. have developed programmable riboregulators to sense microRNAs in mammalian cells.^78^ This approach can be used to detect biomarkers for diseases like cancer. Heo et al. have designed toehold switches to detect pks island mRNAs which are a subgroup of pathogenicity islands in *Escherichia coli*.^70^ This approach can help search for and stabilize single-stranded RNA regions for various applications. Mousavi et al. have developed an electrochemical interface to perform multiplexed detection of antibiotic resistance genes in parallel.^82^ Amalfitano et al. have developed another interface to read output from the toehold sensors using a commercially available glucometer.^83^ These platforms highlight the importance of developing cost-effective, accessible, and user-friendly detection platforms for toehold sensors. However, the clinical samples require a dilution step, which necessitates a preamplification step for the target trigger nucleotide sequence to achieve adequate sensitivity in detecting the target. This preamplification step can be achieved through various isothermal amplification techniques, which will be discussed in detail in the following section.

## Amplification Strategies

**Figure 4:**
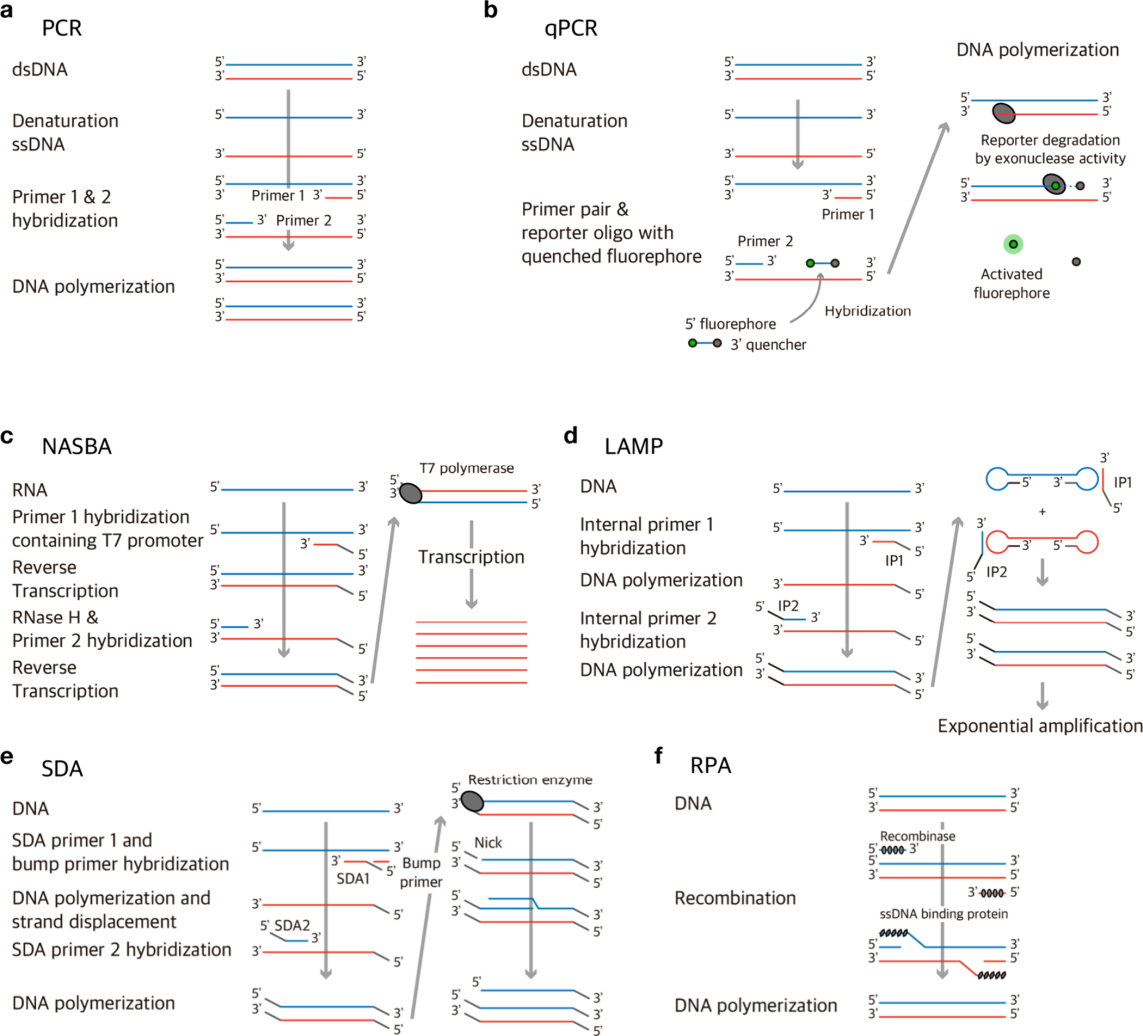
Isothermal nucleic acid amplification strategies. (a) PCR. (b) Probe based real time PCR. (c) NASBA. (d) LAMP. (e) RPA. (f) SDA.

Detection of specific nucleic acids has been extremely valuable in clinical diagnostics,^84^ food safety,^85^ forensic,^86^ and environmental monitoring^87^ applications. A key step in these nucleic acid assays is the amplification step where the assay sensitivity is enhanced by the generation of a large number of target copies, enabling detection and surpassing limitations caused by the nature of the biological samples.^88^ The methods that have a low detection limit and high sensitivity to meet the requirements of reliable detection are generally in combination with PCR (Figure 4a) or its variations (Figure 4b).^89^ Even though PCR revolutionized the molecular diagnostics industry, it limits the implementation of amplification steps in POC devices. The main restraints include thermal cycling steps, being prone to contamination, relatively high expenses, and the need for trained personnel.^90^ Therefore, many isothermal techniques have been developed to implement nucleic acid amplification steps into POC. Each technique offers its advantages as well as its disadvantages. Although its ease of use and effectiveness in amplifying over 10^9^ nucleic acid sequences and identifying bacterial and viral RNA in clinical samples, nucleic acid sequence-based amplification (NASBA) has disadvantages, including the need for specific tools and reagents and the precise adjustment of reaction conditions. Despite its high sensitivity, NASBA is known to be prone to false positives caused by genomic double-stranded DNA, despite its great sensitivity^106^ (Figure 4c). Loop-mediated isothermal amplification (LAMP) has several benefits, including quick detection, cost-effectiveness, a short response time that happens at a steady temperature, and no requirement for complex lab apparatus. However, the difficult work of creating suitable primers could make the LAMP approach difficult and time-consuming to succeed in^107^ (Figure 4d). Recombinase polymerase amplification (RPA) is unique in that it is easy to use, highly sensitive, and selective; it can amplify copies of 1–10 DNA targets in less than 20 min. Rapid amplification eliminates the need for a thermal cycler and works well at room temperature. However, careful primer design might provide extra difficulties and is important. Furthermore, the recombinase uses up all of the ATP it has access to in less than 25 min, which would limit the reaction’s capacity to scale up^108^ (Figure 4f). Strand displacement amplification (SDA) lowers the chance of contamination and eliminates the requirement for a costly thermocycler. It may be considered an easy-to-use alternative for PCR in a number of situations. Long target sequences, however, are difficult for SDA for amplifying, and primer design’s time-consuming process can be considered as a drawback^109^ (Figure 4e).

In these toehold-based switch sensor platforms, isothermal RNA amplification of the trigger region prior to a cell-free system is a necessity. Even though nucleic acid sequence-based amplification (NASBA) is the method used in these articles, assorted substitutes exist.

## Point-of-Care Diagnostics

**Figure 5:**
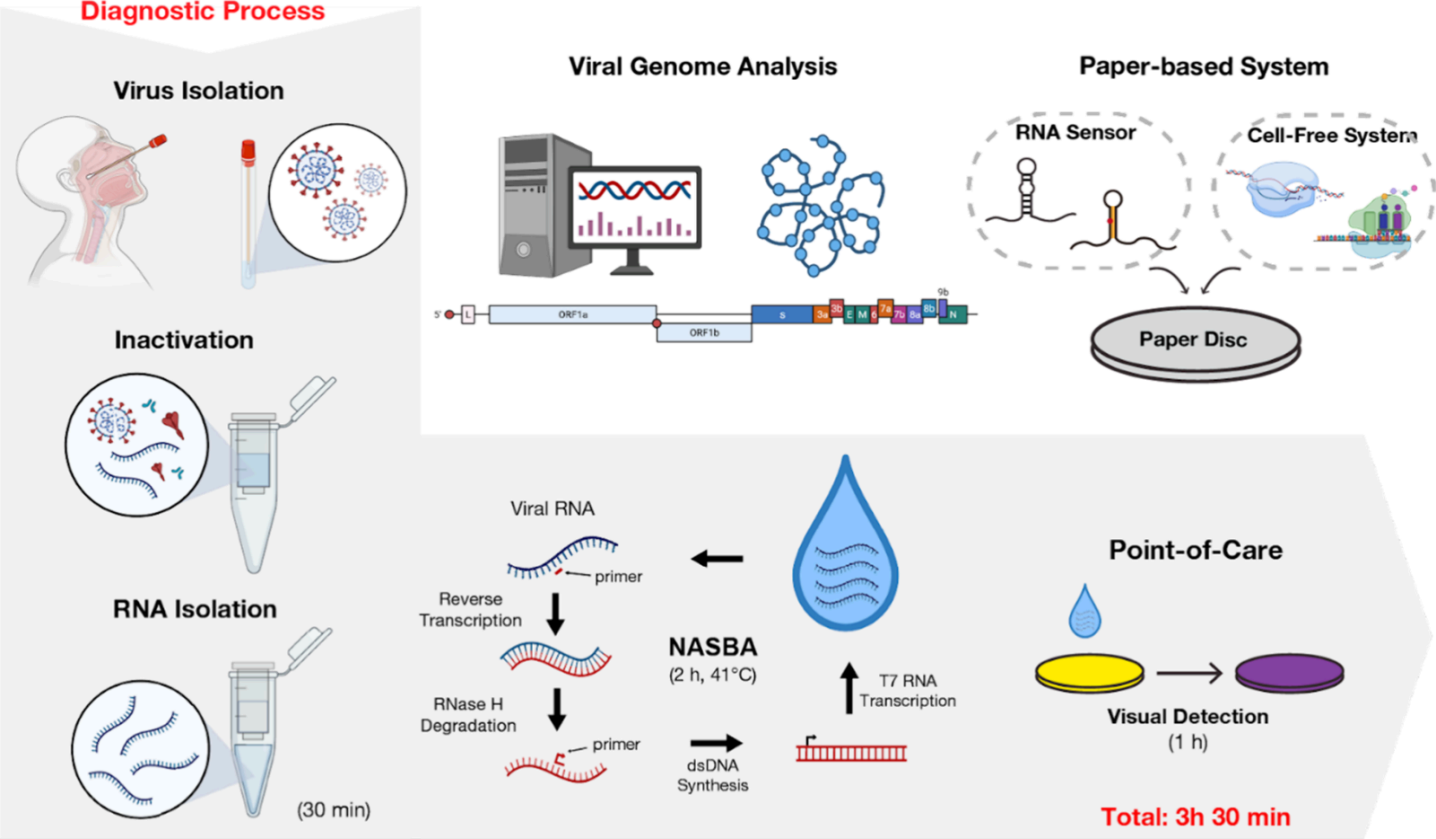
Schematic diagram of the overall POC process through the toehold switch-based technology. The isothermal amplification drawn with NASBA and the detection part with a paper-based assay can be replaced with other techniques. Figure adapted from refs (3) and (121). Copyright 2016 Cell Press. Copyright 2021 Frontiers.

The current pandemic of COVID-19 and other epidemics of the past decade such as Ebola and Zika highlighted the importance of developing elementary POC diagnosis systems.^91^ Current diagnosis systems, such as PCR, require the necessary infrastructure and specially trained personnel to perform the test which may not always be available. To guide and improve the development of POC systems, the World Health Organization (WHO) created the REASSURED criteria (Real-time connected, Ease of specimen collection, Affordable, Sensitive, Specific, User-friendly, Rapid and robust, Equipment-free, Deliverable to end users).^92^ Most of the current widely available systems do not comply with the REASSURED criteria as they require trained personnel or laboratory infrastructure (Figure 5).

RNA-based sensors have certain distinct benefits and drawbacks when compared with other current techniques such as CRISPR or antibody testing. In contrast to CRISPR, CRISPR requires highly experienced workers and advanced machinery. On the other hand, CRISPR technology enables high specificity and multiplexed detection. It may be applied to many other fields outside diagnostics such as gene editing. RNA-based sensors, as opposed to antibody testing, can provide great specificity and early detection of the genetic material of infections. However, antibody tests are often cheaper, quicker, and less sophisticated. They are essential in determining immunological state and prior infection, something that RNA-based sensors are not able to do.^110,111^

Cell-free systems have the advantage of having a simple nature.^93^ They can easily be stored by freeze-drying and, when needed, could be activated with rehydration.^94^ They can be implemented on paper-based or hand-held systems.^95^ In paper-based systems, the sample is dripped onto a special paper that changes its color (based on chemical reactions) or expresses a fluorescent signal that can be measured, based on the presence of the target compound.^96,97^ Paper-based systems that use Toehold switches have been developed to detect Ebola^4^ and Zika^2^ viruses, as well as analyze the gut microbiota.^80^

Hand-held cell-free systems can come in a wide range of different designs, ranging from microfluidic to single reaction chamber ones. They are generally easier to develop compared to paper-based because they have fewer components than paper-based systems and can thus be easily designed to be compatible with a wide range of reactions and analyses, hand-held cell-free systems are more versatile when it comes to adapting to small and portable dimensions for diagnosis and analysis.^111^ Example microfluidic-based systems range from simple ones, such as lateral flow assays (i.e., antibody tests), to more complex designs that work with smartphones to process the data they generate. An example of the latter is a recently developed CD-ROM-based microfluidic device that uses LAMP to detect the presence of the SARS-CoV-2 virus. This system uses a smartphone camera for enhanced fluorescence detection and can yield results in 1 h. Different devices that can be used for output detection are given in Figure 5.

- COVID-19 sensor^45,98^
- Aptamer^99^

## Advantages of Aptamer-Based Cell-Free Riboswitches

Aptamer-based cell-free riboswitches combine the ligand specificity of aptamers with the regulatory function of riboswitches, offering a versatile and customizable platform for ligand-responsive gene expression control. Highly specialized and sensitive RNA sequences known as aptamers can attach to certain targets. Methods to boost aptamer binding affinities and specificities include rational design, guided mutagenesis, and negative or counterselection procedures during the selection process. In order to enhance the functionality of RNA sensors, several optimization studies may be carried out, including affinity tuning and directed mutagenesis. For the purpose of designing more accurate and focused RNA-based detection systems, the use of computational modeling of RNA-ligand binding can help comprehend and forecast interactions between RNA sensors and target molecules.^112^ These riboswitches offer several distinct advantages over traditional protein-based riboswitches and other gene expression control systems.

First, aptamer-based short RNA sequences enable cell-free riboswitches to detect a wide range of target molecules with high affinity and selectivity. Although theophylline has been the most popular target molecule, cell-free riboswitches respond to various targets ranging from molecules and ions to even larger proteins. Second, the customizability of aptamers makes it possible to construct cell-free riboswitches for ligands that do not have known natural riboswitch counterparts. Third, the process of identifying and adapting aptamers for specific ligands is often faster and more amenable to optimization than the discovery of new natural riboswitches. SELEX, the technique used to develop aptamers, can be performed in vitro, allowing for the rapid iteration and selection of well-functioning riboswitches. Additional combinations of aptamers and ligands tailored for cell-free riboswitches will enhance our comprehension of the factors that determine the suitability of an aptamer-ligand pairing for such riboswitches.

## Concluding Remarks and Future Perspectives

In the rapidly evolving landscape of diagnostics, the utilization of RNA-based sensor systems stands out as a versatile and promising avenue, which is especially crucial in addressing the challenges posed by pandemics. The advantages inherent to RNA as a building block for synthetic circuits have paved the way for innovative riboregulators and toehold switches that enable precise and responsive control over gene expression. Despite the challenges associated with building and fine-tuning riboregulators, the past decade has witnessed significant progress in leveraging RNA secondary structure modeling to understand RNA function. The emergence of 3D structures as functional elements adds a new layer of complexity to the design of RNA switches and diagnostic aptamers,^123,124^ with machine-learning-driven tools like FARFAR2^125^ and ARES^126^ offering the potential to design RNA tertiary structures and isothermal amplification regions. This integration of advanced structural insights into sensor design is poised to revolutionize the field of RNA-based diagnostics, enhancing the specificity and sensitivity of the detection platforms.

Looking ahead, the amalgamation of signal amplification strategies, such as NASBA or LAMP, with RNA-based sensor systems holds immense promise in achieving enhanced detection limits, particularly in point-of-care settings. The potential of aptamer-based cell-free riboswitches to extend the capabilities of diagnostic platforms further underscores the versatility of RNA as a critical component of the development of innovative sensing technologies. As the world grapples with the challenges of pandemics and rapid disease outbreaks, the future lies in collaborative efforts at the intersection of molecular biology, engineering, and data science. This interdisciplinary approach will drive the development of RNA-based sensor systems that offer affordable, rapid, and accurate diagnostic solutions, revolutionizing healthcare strategies and bolstering global preparedness for emergent health crises. Ultimately, as we navigate this new age of pandemics, harnessing the power of RNA-based diagnostics is poised to play a pivotal role in safeguarding public health on a global scale.
